# A framework to quantify controlled directed interactions in network physiology applied to cognitive function assessment

**DOI:** 10.1038/s41598-020-75466-y

**Published:** 2020-10-28

**Authors:** Faezeh Marzbanrad, Negin Yaghmaie, Herbert F. Jelinek

**Affiliations:** 1 Department of Electrical and Computer Systems Engineering, Monash University, Clayton, Australia; 2 Department of Biomedical Engineering, University of Melbourne, Parkville, Australia; 3 Department of Biomedical Engineering, Khalifa University, Abu Dhabi, UAE; 4 School of Community Health, Charles Sturt University, Albury, Australia

**Keywords:** Biomedical engineering, Neurophysiology

## Abstract

The complex nature of physiological systems where multiple organs interact to form a network is complicated by direct and indirect interactions, with varying strength and direction of influence. This study proposes a novel framework which quantifies directional and pairwise couplings, while controlling for the effect of indirect interactions. Simulation results confirm the superiority of this framework in uncovering directional primary links compared to previous published methods. In a practical application of cognitive attention and alertness tasks, the method was used to assess controlled directed interactions between the cardiac, respiratory and brain activities (prefrontal cortex). It revealed increased interactions during the alertness task between brain wave activity on the left side of the brain with heart rate and respiration compared to resting phases. During the attention task, an increased number of right brain wave interactions involving respiration was also observed compared to rest, in addition to left brain wave activity with heart rate. The proposed framework potentially assesses directional interactions in complex network physiology and may detect cognitive dysfunctions associated with altered network physiology.

## Introduction

Recent studies in network physiology have advanced our understanding of dynamic interactions, coordination and integration of physiological systems and sub-systems required to maintain optimal collective function^1–3^. However, analysis and interpretation of physiological networks are complicated as the networks are complex due to their multi-component nature, including not only the interactions that form the network but also direct and indirect links between the components, with time-varying strength and direction. The dynamics of brain–heart interactions exemplify such high degree of complexity^4–7^.

Although the interactive effects of cardiac injury on the brain and the effects of brain disorders on the heart have been reported^8–10^, heart-brain dynamics and associated mechanisms are still not sufficiently understood. Furthermore, it has been found that heart-brain communication may differ depending on the different active cortical network components and their associated EEG rhythms when separated into frequency bands^11^ in addition to possible bi-directional respiratory and endocrine influences. The dynamic links between different brain- and heart-rhythms has still remained an open question.

Multiple network interactions associated with cognitive function including memory and attention and the link with respiratory and cardiac function during task activity is one of the main research gaps, studied through cortical and subcortical structure using MRI and corresponding dynamics of brain wave rhythms and activities^12–16^. Cortical network activities including the default mode network and attention network change during various functional brain states from different task-positive states to resting state^12^. For example, alpha band activity (power) associated with the thalamo-cortical default network, has been found to be related to relaxation states, while beta band activity is associated with increased vigilance and attention. The theta and delta power are generally found increased in wake–sleep transitions with reduced vigilance^12,17–20^. However, further investigation is required to better understand these network responses with respect to the lateralisation of cortical activities during rest and cognitive tasks^21^ and more importantly, to extend this to a broader network physiology incorporating cortical, cardiac, respiratory and motor activities that have different bi-directional influences and strength levels depending on the task.

Current tools to assess network physiology rely on various coupling functions. Granger causality (GC) is one of the popular measures to analyze the information flow between time series and has been applied to various physiological data, such as EEG, ECG, heart rate variability (HRV) and blood pressure, during different states such as alertness, drowsiness and sleep^22–24^, undertaking cognitive tasks^25^ or associated with pathological conditions^26,27^. However, Stokes et al. have recently questioned the validity of the use of GC in neuroscience and physiological applications, mainly due to its strong bias or high variance of results, difficulty of interpretation and overlooking the nonlinear interactions and dynamics between systems^28^. Transfer entropy (TE) partially alleviates GC limitations, by providing a model-free generalisation of GC, applicable to nonlinear systems^29^. It has been applied to physiological networks including heart-brain interactions, and has shown its additional value in the analysis of nonlinear interactions^24^. TE is still limited by similar challenges to GC including interpretation, requiring significantly larger data sets and a high computational cost^28^.

A more extensive study of network physiology was performed by Bashan et al. on the interactions of brain, cardiac, respiratory, ocular, and muscle activity, where Time Delay Stability (TDS) was proposed as a novel concept for multi-component systems with various dynamics^2^. It is applicable to diverse physiologic systems with various dynamics over different time scales^11^. The original TDS method however does not distinguish between coupling directions. TDS was further extended in a more generalised time-delay analysis framework, namely delay-correlation landscape, by taking into account the time evolution and time-shift dependencies^5^. Although the sign of time delays indicates the directionality, indirect interactions may confuse any interpretations^30^.

The present work addresses the aforementioned challenges, by proposing a novel framework, as a generalisation of TDS, to assess controlled directed interactions in the network physiology. It is applied to a novel application of identifying inter-hemispheric electrical brain activity measured at the left and right prefrontal cortex and the directional connectivity with cardiac and respiratory rhythms during two cognitive function tests. This method extends the time delay stability concept in network physiology, to reflect directional and pairwise network links, while controlling for the effect of other indirect interactions.

## Results

### Controlled time delay stability to uncover directional primary interactions

The proposed framework, namely Controlled Time Delay Stability (CTDS) stems from TDS, and quantifies the direct dynamical interactions between pairs of nodes in the physiological systems while controlling for indirect interactions. We further visualise the network physiology with nodes showing different physiological systems, connected via directional links indicating their dynamical interactions. This framework demonstrates the directional primary interactions and their changes from one cognitive-physiological state to another. CTDS was evaluated in a simulation example which showed its superior performance compared to the original TDS.

We further demonstrate a practical application of the CTDS for quantifying the interactions between left and right side brain rhythms, heart- and respiration rates, in a cognitive function experiment consisting of an initial rest period, the alertness and attention tasks, the transition between these two tasks and a final recovery period. This experiment resulted in a new discovery of the topology and dynamic interactions of the physiological networks during the cognitive tasks. Results show that the two hemispheres of the brain, the heart and respiratory systems interact in different ways and that the node connectivity, link strength and direction also change during the two different cognitive tasks. This is substantially different from previous studies which only investigated brain structure and dynamics^12–16^, as our framework demonstrates a broader picture showing the role of the heart and respiration in the network depending on the cognitive function state and their relationship to left and right hand side brain rhythms. Generally, we found that compared to rest, the number of active links increases most noticeably during the alertness task within the left side of the brain, and during attention particularly involving the right hand side of the brain in addition to the left hand side. Few but isolated links were also noted during rest, transition or recovery states, which were generally stronger than those in alertness and attention states, however they were inconsistent across individuals.

The network physiology interactions are better characterised with our new CTDS framework, since it not only looks for the time delay at which the dynamics between sub-systems are consistently related, similar to TDS, but also excludes indirect interactions, to focus on the pairwise links. It then also enables identification of the subsystem which follows or precedes the other to assess the directionality. The percentage of time for the observed CTDS indicates the strength of the links (see  “Controlled time delay stability” section). Therefore, the physiological system is modelled as a dynamical network with physiological interactions with directed pairwise coupling if stronger than a significance threshold determined by surrogate analysis.

### Performance of the new framework in simulation

**Figure 1:**
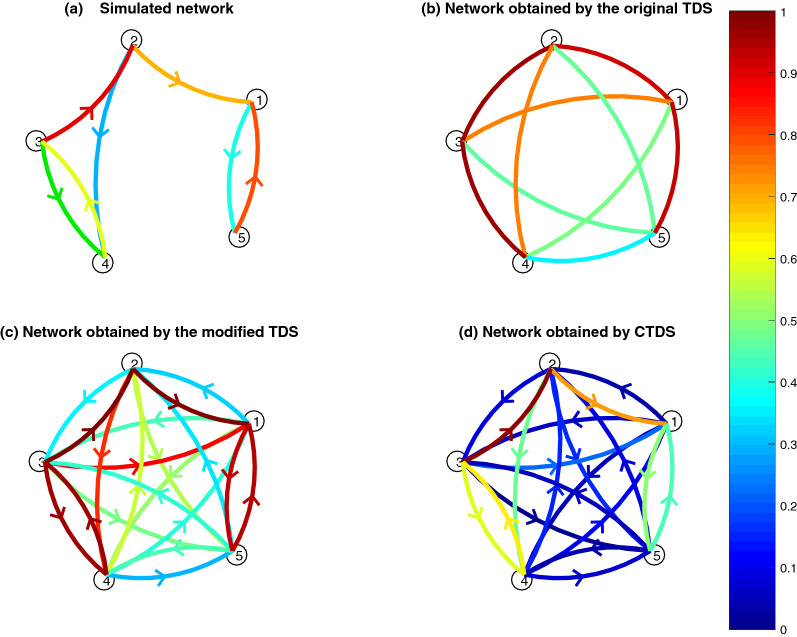
Original and modified TDS, as well as the new CTDS are compared in this simulation study. The strength of the TDS and CTDS links are colour-coded where 10% to 95% range is scaled between 0 and 1 in colour-codes. In the figure, (**a**) shows the original network generated through Eq. (4), with colour-coded coefficients (**b**) illustrates the network determined by the original TDS method proposed in^2^. Although TDS can uncover the couplings between the nodes, it cannot show the direction and whether the couplings are uni- or bi-directional. The network in (**c**) resulted from modified TDS to show the coupling directions only based on the delays and (**d**) shows the network based on the new CTDS method. CTDS not only shows the directions, but also refines the couplings by controlling for indirect relationships and highlighting the primary links. It can better distinguish between primary and indirect links, e.g. the modified TDS found the indirect link from 3 to 1 to be stronger than the primary link from 2 to 4, while in the network derived by the proposed CTDS method, all primary links were stronger than indirect links.

The proposed framework was initially applied to a simulated network as an example, to compare its performance with that of the previous TDS method. The simulation was then extended to various randomly generated networks. As shown in Fig. 1a, and described in “Simulation study” section, and Eq. (4), the methods evaluated a network consisting of five nodes, two bi-directional and three unidirectional links between them, with various coefficients. As shown in Fig. 1b, the original TDS demonstrated stronger links between the nodes with a simulated relationship than other nodes (except one incorrect link between nodes 3 and 1), with average strength being over 70%. However, it does not distinguish between coupling directions. To enable a direct comparison to the CTDS, we modified the TDS method, by specifying the direction of the coupling directly from the sign of stable time delays, i.e. whether each node precedes or follows the other. Then we compared this modified TDS method with the new CTDS (Fig. 1c,d) and found a better performance by the proposed CTDS method.

**Figure 2:**
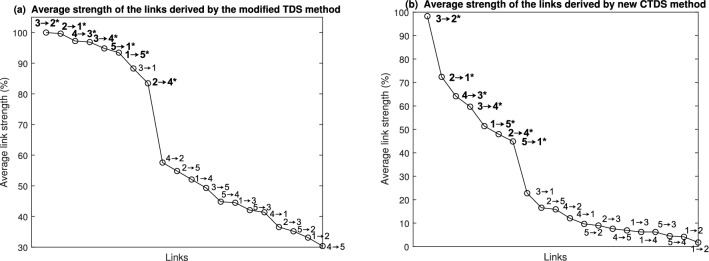
Average strength of the links for the simulated network in Fig. 1a is illustrated for the links derived by (**a**) modified TDS which indicates coupling directions, and (**b**) the new CTDS framework. The original TDS is not presented as it does not provide any information about the coupling direction. The primary links (as shown in Fig. 1a) are in bold and marked with ‘*’. The figure indicates that the proposed CTDS method can distinguish better between primary and indirect links e.g. using a threshold of 40%, while the modified TDS, resulted in a stronger indirect link (3 to 1) than the primary one (2 to 4).

More specifically, as shown in Fig. 2, one indirect link (3 to 1) is found to be stronger than a primary link (2 to 4) by the modified TDS, while CTDS consistently finds all primary links stronger than non-primary links. It therefore provides a better distinction between primary and non-primary links.

The simulation was further extended by generating random networks with various numbers of nodes from 4 to 8. For each simulated network, various subsets of two nodes were randomly selected to have uni- or bi-directional links, with random coefficients in the range of $$-1$$ to 1, multiplied by an exponential decay term to decline at a rate proportional to the lag between them. The time series were generated with five different amplitudes of the additive Gaussian noise ($$k=10^{-2}, 10^{-2}, 1, 10^{1}, 10^{2})$$. The results were evaluated for 10 randomly generated networks for each selection of noise amplitude and number of nodes, as summarised in Table 1. The table shows that CTDS finds the actual primary links to be significantly stronger than non-primary/non-existent links, for all network sizes and noise levels, as shown by Mann Whitney Wilcoxon (MWW) *p*-values $$<0.05$$ and large difference between average strength of primary links and others (denoted by $$\Delta $$). Athough TDS resulted in significantly stronger primary vs other links for smaller networks ($$\hbox {n}=4$$, 5, 6), it failed to achieve that for larger networks, particularly for higher levels of noise, where there is a higher chance of indirect pathways from one node to the other. The new CTDS method addresses this issue by controlling for other nodes when computing the link between each pair of nodes, to avoid the indirect interactions.

**Table 1:** Comparison of the strength of actual and non-existent links estimated by the modified TDS and CTDS.

	$\hbox {k}=0.01$		$\hbox {k}=0.1$		$\hbox {k}=1$		$\hbox {k}=10$		$\hbox {k}=100$	
	TDS	CDTS	TDS	CDTS	TDS	CDTS	TDS	CDTS	TDS	CDTS
**n = 4**										
MWW (p)	0.0057	0.0156	0.0020	0.0004	0.0001	0.0004	0.0020	0.0009	0.0005	0.0129
Mean $\Delta$	24.58	27.62	27.98	35.86	34.78	28.13	25.81	31.18	32.82	20.67
Min $\Delta$	1.31	0.00	0.00	18.39	25.07	5.75	0.00	9.05	12.60	− 0.69
Max $\Delta$	58.62	52.01	40.34	66.78	52.30	58.85	42.53	55.60	53.91	55.24
**n = 5**										
MWW (p)	0.0270	0.0009	0.0057	0.0004	0.0444	0.0070	0.0036	0.0001	0.0755	0.0002
Mean $\Delta$	17.85	31.83	19.20	30.37	22.72	28.76	26.11	32.64	18.95	31.06
Min $\Delta$	− 0.02	13.03	− 1.16	10.88	0.00	8.51	0.35	23.61	− 4.30	14.35
Max $\Delta$	25.99	51.02	33.16	52.31	48.63	50.34	44.63	47.68	37.73	59.08
**n = 6**										
MWW (p)	0.0247	0.0005	0.0002	0.0001	0.0156	0.0011	0.0206	0.0011	0.0106	0.0002
Mean $\Delta$	17.40	34.29	23.70	35.06	21.16	29.67	16.44	27.64	17.78	33.00
Min $\Delta$	0.00	20.27	12.61	14.08	0.77	3.83	0.00	8.02	1.54	18.03
Max $\Delta$	35.72	50.29	32.42	54.21	34.98	48.24	32.15	44.04	28.86	45.17
**n = 7**										
MWW (p)	0.0562	0.0086	0.2853	0.0057	0.2603	0.0002	0.1132	0.0106	0.0606	0.0009
Mean $\Delta$	11.71	21.39	9.46	25.36	10.87	26.88	9.84	18.74	12.76	24.29
Min $\Delta$	0.02	6.70	− 0.46	5.62	− 4.22	17.90	0.00	8.02	0.41	13.10
Max $\Delta$	28.47	29.64	22.29	46.48	29.26	44.79	22.18	31.27	24.75	38.12
**n = 8**										
MWW (p)	0.0270	0.0007	0.2853	0.0378	0.0929	0.0188	0.1537	0.0036	0.2028	0.0270
Mean $\Delta$	12.79	26.31	4.39	18.17	10.81	20.45	10.11	25.67	6.40	16.27
Min $\Delta$	− 1.57	9.53	− 6.46	3.29	0.13	6.65	− 2.29	12.05	− 1.59	3.53
Max $\Delta$	25.34	41.11	22.70	31.15	28.08	37.75	21.31	32.12	19.93	35.42

Mann Whitney Wilcoxon (MWW) *p*-values were found for comparison of the average strength of primary versus non-existent/non-primary links for randomly generated networks with $\hbox {n}=4$ to 8 number of nodes and $\hbox {k}=0.01$ to 100 noise amplitude. Mean, Min and Max of $\Delta$ denote mean, minimum and maximum values of strength difference ($\%$), i.e. average strength of primary links − average strength of non-primary/non-existent links. The results show that CTDS found significantly stronger links for primary links than other links (MWW ${p}<0.05$ and positive and larger difference $\Delta$). TDS resulted in some non-significant differences particularly for larger networks ($\hbox {n}=7,8$) with higher level of noise and smaller difference of average link strength.

**Figure 3:**
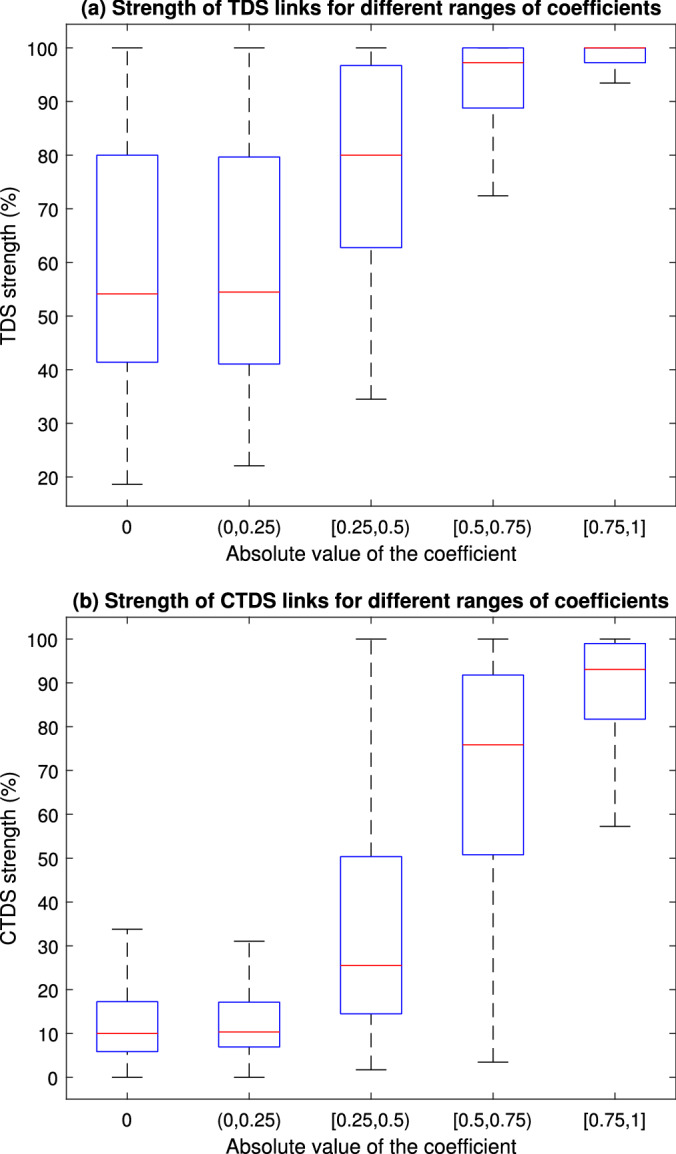
The link strength obtained by (**a**) the modified TDS and (**b**) CTDS, are shown for various coefficients of links for randomly generated networks with 6 nodes and a range of noise amplitude ($\hbox {k}=0.01$ to 100), excluding the outliers. Comparison of link strength found by the modified TDS and CTDS shows that both methods fail to effectively differentiate weak links (coefficient $<0.25$ for this example) from non-existent/non-primary links (with zero coefficients). For larger coefficients ($\ge 0.25$), CTDS can better differentiate various levels of interactions which are generally stronger than the links with coefficients $<0.25$.

The link strength values derived by the modified TDS and CTDS were also evaluated for various coefficients of simulated links for simulated networks with $\hbox {n}=6$ nodes and a range of noise amplitude ($\hbox {k}=0.01$ to 100). Figure 3 compares the link strength found by the modified TDS and CTDS for different ranges of coefficients of simulated networks. Results show that both methods cannot effectively differentiate weak links (coefficient $<0.25$ for this example) from non-existent/non-primary links. However for larger coefficients, CTDS can better differentiate various levels of interactions from each other and from weak and non-existent/non-primary links, while there are larger overlaps between TDS strength of links with different levels of coefficients. Generally TDS tends to detect higher levels of strength, since it does not control for indirect interactions between nodes. CTDS link strength was also better correlated with the magnitude of coefficient. Pearson correlation test results comparing the model link coefficient (magnitudes) versus the link strength inferred by TDS and CTDS, showed a stronger correlation between CTDS links and coefficients ($\rho = 0.73$, $p < 0.0001$) than between TDS links and coefficients ($\rho =0.53$, $p < 0.0001$).

### Network physiology changes with cognitive function

**Figure 4:**
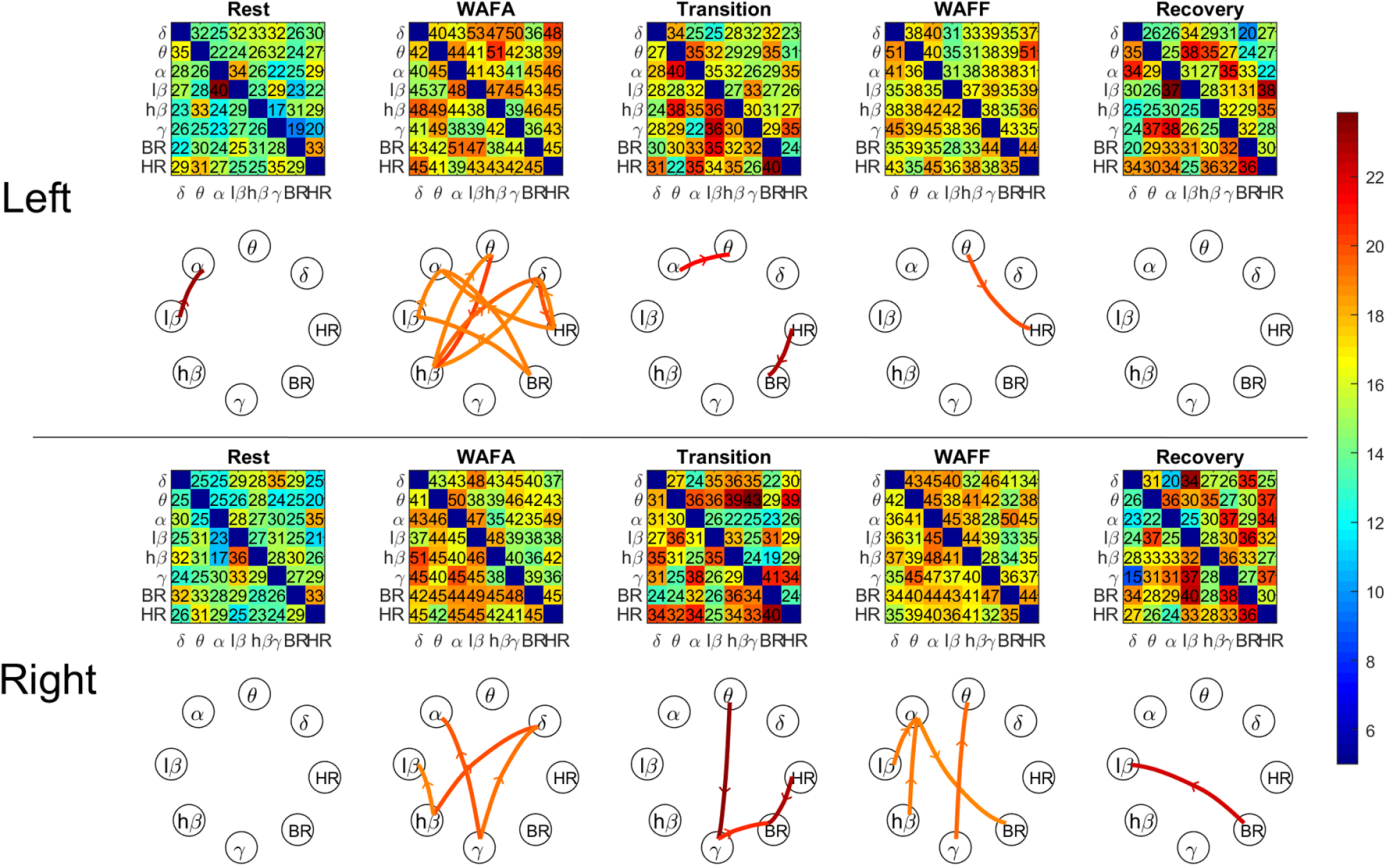
Group-averaged CTDS coloured matrices and corresponding network physiology of left- and right-side brain rhythms, heart and breathing rates, interactions during initial rest; WAFA alertness test; transition (rest); WAFF attention test and final recovery (rest). Each matrix element colour-maps the average strength of the CTDS (the fraction of time with stable delay over the total phase duration) for each pair of physiological systems. The coloured matrices show the strength averaged over all subjects during each phase. The number in each matrix element is the percentage of cases with significant links (CTDS strength > threshold) out of the total number of cases ($N=110$). The threshold was determined by surrogate analysis ($k=19$, see “CTDS analysis and surrogate test” section and Fig. 5). The links are displayed in the network graph with arrows, only if CTDS strength in that specific direction is greater than the threshold ($k=19$) commonly for more than $40\%$ of cases. This representation resulted in a fewer number of consistent links during the rest phase than for the attention and alertness phases, although the isolated links during rest (left side) or transition and recovery (right) appear stronger than those during cognitive tasks, as also shown in Fig. 6.

In our experimental cognitive function study we found that the network physiology changed from initial rest to the WAFA alertness test (Schufried GmbH, Vienna), followed by another rest (transition) period, then to the WAFF focused attention test (Schufried GmbH, Vienna) and the final rest (recovery), with the left and right hemisphere locations having different signal characteristics (Fig. 4).

The initial surrogate analysis, provided an appropriate threshold level to identify significant links.

**Figure 5:**
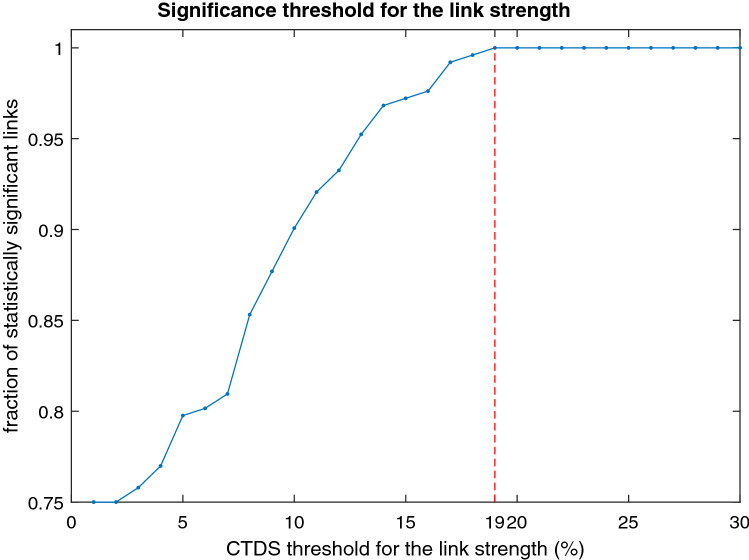
Surrogate test results indicated that a threshold set at 19% for link strength (shown with the vertical dashed line) allows only significant links with relevant physiological information to be reliably included in the analysis.

As shown in Fig. 5, $k=19\%$ is the threshold level above which, 100% of links are statistically significant. This significance was determined by the Student t-test showing that the links are significantly stronger (*p*-value $<10^{-3}$) than the surrogate ones (i.e. the same systems but for different subjects). More details about the surrogate test can be found in “CTDS analysis and surrogate test”.

**Figure 6:**
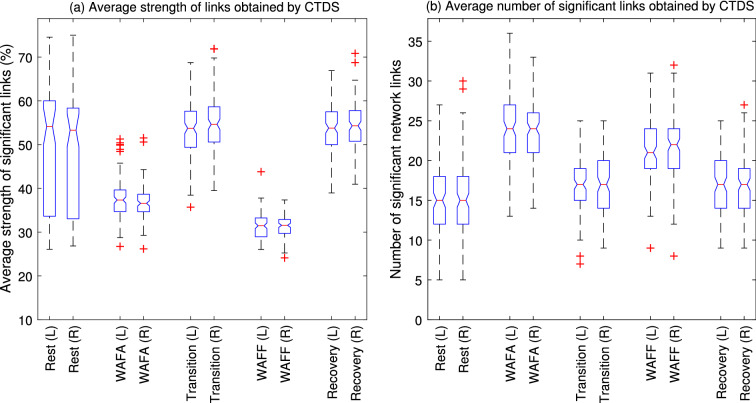
(**a**) The boxplots show the distribution of the CTDS strength of significant links over 110 subjects. A larger variability range for the link strength for subjects at rest, and lower link strengths during the WAFF test were observed. Overall, from Fig. 6b and this figure, there are fewer, but stronger links for rest, compared to WAFA and WAFF. According to the MWW test results, the strength of the links significantly differs between WAFA and all other states (${p}<0001$), and between WAFF and all other states (${p}<0.0001$) for both sides of the brain. No significant difference was found between the left and right sides of the brain during the same cognitive state (WAFA and WAFF). The strength of the links associated with right side during the rest phase was significantly different from both transition and recovery (same side), but it was not significant for the left side. (**b**) The boxplots show the distribution of the number of significant links by CTDS over all subjects ($N=110$). It shows a higher number of links during the two cognitive tasks compared to the resting phases. According to MWW test results, the number of links were significantly different between rest and all other states (${p}<0.005$), WAFA and all other states (${p}<0.005$), WAFF and all other states (${p}<0.005$). No significant difference was found between transition and recovery. No significant difference was found between the links associated with the left and right hand sides of the brain during the WAFA but were found for WAFF attention test (${p}<0.05$).

Figure 4 indicates that during rest, only one significant link among brain waves (delta, theta, alpha and beta) was observed for 40% of cases, namely from the $lower \beta$ (13–18 Hz) to $\alpha$ (8–13 Hz) on the left side of the brain. This link weakens during subsequent phases, but becomes stronger again during the final rest (recovery) phase, although only for 37% of cases, hence not shown in the graph. On the right side no link reached significance during the initial rest phase, or the links were inconsistent across individuals, i.e. not significantly stronger for over 40% of cases. For the current experiments, the number of significant links during the initial rest period on both right and left hand sides of the brain, were generally significantly lower than all other states (MWW test ${p}<0.005$) as shown in Fig. 6b. These links are however stronger than the links during the WAFA and WAFF states, as shown in Fig. 6a.

The system interactions during the rest phase were less consistent across participants, as they also resulted in a larger variability in the link strengths, compared to other phases (Fig. 6a). This could be due to an arbitrary cognitive state of the participant during the starting phase, since they were not performing any specific task.

During the WAFA alertness test, more links became stronger than the threshold, compared to the initial rest phase as shown in Figs. 4 and 6b. Although the number of significant links did not differ between the right and left sides of the brain, they were the highest among all states and those links connected different subsystems on the left and right sides. Of interest is that the brain rhythms on the left side were more engaged with heart rate and breathing than the brain rhythms on the right side, particularly during alertness. Also different intra-hemispheric connection patterns were found between brain wave rhythms during the WAFA test, e.g. (4–8 Hz) interacted bi-directionally with higher $\beta$ (18–30 Hz) only on the left side whereas significant links from $\gamma$ (30–45 Hz) to $\alpha$ and $\delta$ (2–4 Hz) were only observed on the right side. This suggests that the different functions of the left and right hand side of the brain are reflected by different brain wave rhythm connections especially as seen in the current experiments during the WAFA alertness test.

The WAFA test was followed by a rest phase (transition) during which the number of significant links were significantly lower (but stronger) than observed during the alertness phase, but higher than the initial rest phase (Fig. 6b). After the transition rest phase, the WAFF focused attention test was performed. The coloured matrices in Fig. 4 indicate that there were many links between the brain rhythms on the left brain, heart rate and respiration which more than ($40\%$ of) participants had in common. However, they did not reach the threshold for significance in this set of experiments. One exception was the link from (4–8 Hz) to heart rate. A higher number of significant links were found for the right side than the left, as shown in Figs. 4 and 6b and between different brain wave rhythms. Overall, the number of significant links were higher during the WAFF test, compared to the three rest states, but smaller than those during the WAFA test. The strength of the links during the WAFA test were the lowest of all phases. The final rest (recovery) phase consisted of approximately the same number of significant links as the transition rest state between the WAFA and WAFF tests. Overall, we found a higher percentage of participants with links in common during the WAFA and WAFF tests as shown in Fig. 4.

Cardio-respiratory coupling, which has been extensively studied during rest periods was not evident during the initial rest phase, but became stronger during subsequent states, although only significant during the transition phase. This suggests that cardio-respiratory coupling changes with cognitive function states and may also be sensitive to influences such as stress or expectations during rest.

**Figure 7:**
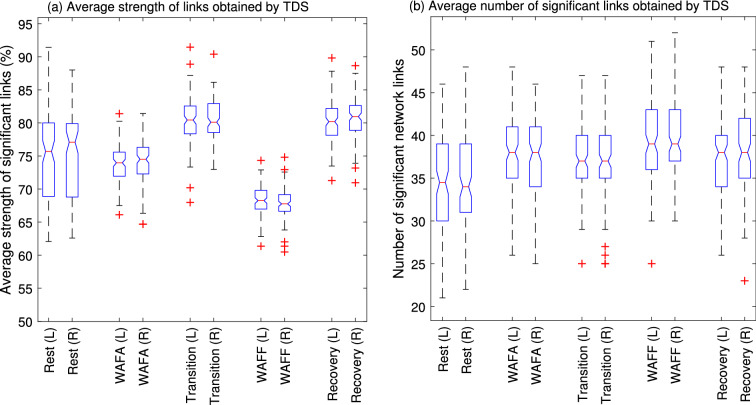
(**a**) The boxplots show the distribution of the TDS strength of significant links over 110 subjects. Generally TDS strength were higher than that of CTDS, for most links, with less variability between states, compared to CTDS. TDS also found similar patterns for changes in the link strength between states. It also found a larger range variability during rest, similar to CTDS. Consistent with CTDS results but with less significance, there are fewer, but stronger links for rest, compared to WAFA and WAFF. According to the MWW test results, the strength of the links significantly differs between WAFA and all other states (*p* < 0001), except for the rest (left-side), and between WAFF and all other states (*p* < 0.0001) for both sides of the brain. No significant difference was found between the left and right sides of the brain during the same cognitive state, except for recovery (*p* = 0.04). (**b**) The boxplots show the distribution of the number of significant links over all subjects (*N* =110) found by the modified TDS. Generally higher number of links was found by TDS than CTDS. However, the trend of changes in the number of links across states is similar to that of CTDS results. It shows slightly higher number of links during the two cognitive tasks compared to the resting phases. According to MWW test results, the number of links were significantly different between rest and all other states (*p* < 0.005), WAFA and WAFF (*p* < 0.005), WAFF and all other states (*p* < 0.005). No significant difference was found between WAFA, transition and recovery. No significant difference was found between the links associated with the left and right hand sides of the brain.

We also applied the modified TDS to the physiological data for comparison. As shown in Fig. 7. Comparing Figs. 6 and 7, very similar patterns of changes in network physiology are observed with a lower level of significance, i.e. higher number of links during cognitive states but with less strength. However, similar to our simulation results, TDS found a larger number of strong links, making it difficult to visualise and interpret. Hence the new CTDS provides a more natural interpretation of the physiological changes of cortical processing.

## Discussion

The proposed CTDS framework extends the concept of TDS, to address its limitation in distinguishing between different coupling directions. CTDS enables quantification of directional interactions. Unlike TDS, CTDS also quantifies the coupling links between pairs of systems while controlling for indirect links. In this study CTDS quantified the coupling between pairs of specific brain wave frequencies on the left and right side of the brain (sensors placed at Fp1 and Fp2) as well as respiratory and cardiac systems while controlling for indirect links with other systems in the network. It can identify robust interactions between physiological systems in both directions and allows investigation of their stability across participants during different physiological/cognitive states.

Besides the theoretical formulation of CTDS and demonstrating its desired performance during simulation experiments, the major part of our study aimed to assess the network physiology measured as links between systems during two cognitive function states, namely attention and alertness. We found that although TDS resulted in similar patterns to those obtained by CTDS, TDS found a larger number of strong links, possibly including non-primary links and those resulting from covariates, which makes it difficult to visualise, distinguish and interpret the interactions. Better performance of CTDS, particularly for larger networks, is however achieved at the cost of extra computations. Equation (2) shows that for each pair of nodes (*x* and *y*), additional terms, namely, $\rho _{xz}$ and $\rho _{yz}$ cross-correlations, are computed for CTDS while controlling for one extra node (*z*). In comparison, TDS only requires computation of one cross-correlation term ($\rho _{xy}$). The level of computation grows for larger networks. However both methods can be implemented in computationally efficient ways by vectorisation methods^31^.

During the changes between cognitive function phases alternating with rest phases, we identified varying network patterns. A significantly higher number of network links were observed during alertness and attention states, compared to the rest phases. This shows the higher level of connectivity between brain and cardio-respiratory rhythms during cognitive tasks, which was not possible in previous studies on cognitive function as they only focused on brain rhythms^12–16^.

While the overall number of significant links did not differ significantly between the left and right sides of the brain, ipsilateral network evaluation showed different patterns of interactions for the left and right brain rhythms and cardio-respiratory coupling for the WAFA and WAFF cognitive tasks. More specifically during the alertness task (WAFA), there were more interactions on the left-hand side of the brain, which also engaged the heart and breathing rhythms, which was not the case for the right side. During the attention test (WAFF) there were more interactions on the right side of the brain, also affecting the breathing rate, rather than on the left side where only theta wave was found to interact with the heart rate.

Functional connectivity may play an important role for optimising task efficiency and task integration within or between functional networks at rest or during task conditions^32^. Lateralisation of brain function has been known for some time with the left hemisphere being more associated with language and motor function, whereas the right hemisphere is characterised by visuospatial attention^33^. Lateralisation may therefore be dependent on specificity of task or whether functional integration is required. More recent findings further suggest that the left hemisphere more strongly interacts within the same hemisphere, whereas the right hemisphere interacts with both hemispheres^33^. Our results agree with previous work proposing a lateralisation based on functional connectivity density mapping of long-range connectivity or functional integration and short-range connectivity implicated in functional specialisation^34^, which may be now extended to include networks other than cortical networks such as the cardiac and respiratory systems.

Our results established that the attention task had stronger links between right brain wave rhythms with some cross-over to the left hemisphere whereas for alertness, the links were stronger on the left side. Importantly however are our findings that the type of links either between cortical EEG rhythms only or extending to respiratory and cardiac rhythms differ between tasks and that these differ to resting periods. Our results further support the proposal that lateralisation may be an advantage for dual tasking by enhancing brain efficiency during cognitive tasks and suggests that Alertness and Attention are subtly different types of cognitive processes that are bidirectionally linked to diverse physiological networks^35–37^.

Brain processes of working memory involve oscillatory activities at multiple frequencies in local and long-range neural networks. The relevance of changes in the magnitude of cortical oscillations has also been reported to be modulated by the demands of a specific task^38^. Alpha wave magnitude or power indicates a state of desynchronisation in which local neural assemblies become increasingly independent in preparation for a subsequent active process^39^. Cortical and subcortical coupling with ANS function has been studied primarily in psychopathology in association with the limbic system^40^. Our findings now extend this to cognitive function associated with the prefrontal cortex and defines not only laterality but directionality as well.

Overall, during the WAFA and WAFF the coupling patterns were more consistent across the participants. Although the couplings during the rest phases were stronger compared to the cognitive task phases, there were less consistent and more isolated across the participants. This might be explained by the well-defined tasks which were consistently performed by all individuals during WAFA and WAFF tests, rather than being in a more general and arbitrary rest state. The observed network topology and interactions which changed for the WAFA and WAFF, provides new insights into the collective role of physiological systems during various cognitive states and the importance of network physiology as a model in health and disease.

Although we focused on a specific alertness and attention task in this study, the proposed framework can enable future investigations on directional physiological interaction during other cognitive functions and beyond. Only healthy participants with no known or reported cognitive dysfunction or general health issue were included in the current experiments. However, our method is potentially useful in clinical practice where the network physiology is affected by individual system malfunction or altered interactions for cases with cognitive impairment or other pathological conditions. Future studies in our laboratory are investigating the use of this framework in assessing the dynamical interactions of the physiological systems for cases with cognitive dysfunction and effectiveness of treatment. On a broader perspective, our framework is applicable to a wider range of applications to explore the directional interactions of complex, interdependent and dynamic networks, beyond the physiological and clinical implications.

## Methods

### Modified time delay stability

The proposed framework extends the TDS method for integrated physiological systems interacting with a range of time delays^2^. TDS quantifies stability and strength of interactions with a stable time lag between the signals of the coupled systems. It determines the coupling strength by evaluating the periods of constant time delays. In brief, it quantifies the interaction between two physiological systems *X* and *Y*, through output signals *x* and *y* by first windowing them into $N_L$ overlapping segments *v* of length *L*, and normalising (subtracting the mean and dividing by the standard deviation). Then it finds the cross-correlation function for each segment, accounting for a time delay $\tau$:

1 $$\begin{aligned} \rho _{xy}^{(v)}(\tau )={1/L}\sum _{i=1}^{L} x_{i+(v-1)L/2}^{(v)} y_{i+(v-1)L/2+\tau }^{(v)} \end{aligned}$$

The delay $\tau =\tau ^v_0$ resulting in maximum absolute value of the cross-correlation function is then found in each segment. Relatively constant delay in a defined limited range is assumed to indicate stable coupling between two signals, while its large variability represents absence of stable coupling. Overall stability is then defined as at least four out of five consecutive segments with stable time delays, namely Time Delay Stability (TDS), where longer period of TDS is equivalent to more stable coupling. Strength of the coupling is defined as the percentage of time with observed TDS, with a threshold level determined through surrogate analysis.

TDS method in its original form does not distinguish between the coupling direction, i.e. whether each subsystem proceeds or follows the other^2^. Since in this work a directional method is proposed, we modified the original TDS to allow for directionality and to be able to compare the TDS and the new CTDS frameworks. To this aim, we assumed the time delay $\tau$ to be a positive value, and found the stable time delays maximizing $\rho _{xy}^v$ and $\rho _{yx}^v$ (in Eq. (1)), corresponding to system *Y* following or preceding system *X*, respectively.

### Controlled time delay stability

One of the main contributions of the proposed CTDS framework is to distinguish between primary (direct) links and non-primary (indirect) ones in a complex network, which isolates the directional links between each pair of systems, while controlling for the effect of other nodes. Therefore, it further refines the actual delay and direction corresponding to each link rather than being the delay incurred via indirect link through different pathways involving other nodes. This was achieved by finding a stable delay which maximises the partial cross-correlation of each pair of nodes, while controlling for other systems in the network.

If there is an additional system *Z* with output signal *z*, interacting with the other two systems *X* and *Y*, it can influence their coupling via indirect pathways. In order to account for this indirect link, the network interactions should be corrected for the effect of *z* on the cross-correlation between *x* and *y*, the output signals associated with *X* and *Y*. Based on the concept of partial cross correlation^41,42^, the partial cross correlation can be written as:

2 $$\begin{aligned} \rho _{xy|z}(\tau )=\frac{\rho _{xy}(\tau )-\rho _{xz}.\rho _{zy}(\tau )}{\sqrt{1-\rho _{xz}^2 ~} \sqrt{1-\rho _{zy}^2(\tau )}} \end{aligned}$$

Assuming that there are *N* additional systems $Z_1, Z_2,\ldots , Z_N$ interacting with *X* and *Y*, the partial cross-correlation is extended to higher order partial cross-correlation, by controlling for *N* other output signals $z_1, z_2,\ldots , z_N$. By recursion, the $N^{th}-$order partial cross-correlation is as follows^43^:

3 $$\begin{aligned} \rho _{xy|z_1, z_2,\ldots , z_N}(\tau )=\frac{\rho _{xy|z_1, z_2,\ldots , z_{N-1}}(\tau )-\rho _{xz_N|z_1, z_2,\ldots , z_{N-1}}.\rho _{z_Ny|z_1, z_2,\ldots , z_{N-1}}(\tau )}{\sqrt{1-\rho _{xz_N|z_1, z_2,\ldots , z_{N-1}}^2 ~} \sqrt{1-\rho _{z_Ny|z_1, z_2,\ldots , z_{N-1}}^2(\tau )}} \end{aligned}$$

This coefficient can be calculated through a regression procedure, noting that the partial cross-correlation coefficient $\rho _{xy|z}$ is the correlation coefficient between the residuals of *x* and *y* signals after the regression on control signal *z*. The partial cross-correlation function is obtained for each $N_L$ overlapping normalised segments *v* of length *L*, accounting for a time delay $\tau$. Then for each segment *v*, the delay $\tau ^v_0$ which maximises the function $\rho _{xy|z_1, z_2,\ldots , z_N}(\tau )$ is found. Similar to TDS, the relatively constant delay in a defined limited range indicates stable coupling, and overall stability requires as least four out of five consecutive segments with stable time delays. The threshold level is determined through surrogate analysis for each application, explained in “CTDS analysis and surrogate test”.

### Simulation study

The performance of the new CTDS method was evaluated and compared to TDS, in two simulation studies. In the initial simulation, as shown in Fig. 1a, the generated network consisted of five nodes, interconnected through seven directed links. The signals $x_1, x_2,\ldots , x_5$ correspond to nodes 1 to 5, contained 100 samples each and were generated as follows:

4 $$\begin{aligned} x_1(i)&=0.7x_2(i-1)+0.8x_5(i-3)+w_1(i)\nonumber \\ x_2(i)&=0.9x_3(i-1)+w_2(i)\nonumber \\ x_3(i)&=0.6x_4(i-3)+w_3(i)\nonumber \\ x_4(i)&=0.3x_2(i-2)-0.5x_3(i-2)+w_4(i)\nonumber \\ x_5(i)&=0.4x_1(i-3)+w_5(i)\nonumber \\& \text {where } i=1, 2,\ldots , 100. \end{aligned}$$

$w_1, w_2,\ldots , w_5$ were Gaussian noise samples taken from normal distribution.

Original TDS, modified directional TDS (see “Modified time delay stability” section), and the new CTDS framework were applied to the simulated network in MATLAB, and the results were shown in Fig. 1.

The simulation was then extended by randomly generating networks with $n=4$ to 8 nodes and five different noise amplitudes. For each selected number of nodes and noise amplitude, 10 sets of time series were randomly generated. For each node, the time series were generated as follows:

5 $$\begin{aligned} x_i(t)=\sum _{d=1}^D W_{i,j,d}e^{-0.1d}x_j(t-d)+w_i(t) \end{aligned}$$

where each pair of nodes (*i* and *j*) were randomly assigned coefficients ($W_{i,j,d}$) which can be either zero (i.e. no link) or nonzero. The nonzero coefficients were randomly generated from real positive or negative values from − 1 to 1. The coefficients were also multiplied by an exponential decay term to decrease at the rate of $0.1 \times$ the lag between time series. $w_i$s were Gaussian noise samples taken from normal distribution with amplitude of $k=0.01$, 0.1, 1, 10 or 100. Modified TDS and CTDS were applied to the generated time series and the strength of the primary links ($W \ne 0$) and non-primary links ($W = 0$) obtained by two methods were compared for the networks with different number of nodes, noise amplitudes and coefficients.

### Cognitive function experiment

The proposed framework was applied to a cognitive function experiment, in which the interactions between brain rhythms, heart rate and breathing rate were investigated.

#### Data collection and procedure

The study recruited 110 participants at the Charles Sturt University Diabetes Complications Health Research Clinic. Written informed consent was obtained from each participant, following an information session with each potential participant having the opportunity to ask questions of the principal investigators. Ethics approval was obtained from the Charles Sturt University Human Ethics Committee and the study was conducted in accordance with the World Medical Association Declaration of Helsinki (Approval Number 2006-042).

The participant had an initial rest for an average 4.4 (± 3.6 std) minutes. Then they performed the WAFA intrinsic-visual test of alertness. More specifically, the participants should notice a circle appearing and disappearing; and must react as quickly as possible by pressing a button each time. The test took an average of 4.6 (± 0.4) minutes, and was followed by another rest (transition) period of average 2.1 (± 0.4) minutes. After this rest period, the WAFF test of focused attention was performed, where participants were shown a square which appeared and disappeared, while appearing sometimes lighter before disappearing, and other times remaining dark. The participant had to watch closely and press a button as quickly as possible after the square appeared and then became lighter twice in succession. The WAFF test took an average of 9.9 (± 1.6) minutes and was followed by a final rest (recovery) of average 2.1 (± 0.1) minutes. While performing these tasks, a single channel ECG, and two channel EEG from right and left frontal sites were recorded, using Biopack Student Lab 3.7.7, and sampled at 1000 samples per second.

#### Pre-processing

ECG signals were initially processed by a band-pass filtering (cut-off frequencies: 5 Hz and 45 Hz) for noise and artefact removal, and QRS locations were identified to find the heart rate using a modified Pan and Tompkins method^44^. Respiration rate was derived from the ECG using respiratory sinus arrhythmia^45^.

Automated Wavelet Independent Component Analysis (WICA) was used to remove artefacts such as cardiac, muscle, eye movement and transmission line interference from EEG recordings^46^. For this purpose, EEG Rhythms were extracted through Discrete Wavelet Transform (DWT), and decomposed into 8 levels using a Daubechies 4 wavelet. Rènyi Entropy and Kurtosis of each Wavelet Component were calculated and normalised, and those exceeding a threshold were marked as critical and Independent Component Analysis (ICA) was applied to them, to extract Wavelet Independent Components (WICs). The WICs were segmented into smaller windows, and those with Kurtosis or Rènyi Entropy exceeding the threshold in more than one fifth of the signal, were discarded. The edited critical wavelet components were then reconstructed by applying the inverse ICA and restored into the original wavelet dataset. The denoised EEG signals were obtained by taking the inverse DWT of the edited Wavelet Components. The following brain rhythms were then extracted using Welch’s power spectrum: delta ( $\delta$) at 2–4 Hz, theta ($\theta$) at 4–8 Hz, alpha ($\alpha$) at 8–13 Hz, lower beta ($l\beta$) at 13–18 Hz, higher beta ($h\beta$) at 18–30 Hz and gamma ($\gamma$) at 30–45 Hz.

#### CTDS analysis and surrogate test

The processed set of physiological signals (as described above), were windowed into 30-second segments with 50% overlap, and then normalised to perform CTDS analysis (see “Controlled time delay stability” section).

The threshold for stable time delays was determined through surrogate testing, to find a threshold for detecting physiologically relevant interactions. The surrogate test was performed by applying CTDS to the pairs of physiological signals from different subjects. For 100 surrogates, a student t-test was performed to determine the statistical significance comparing the distribution of CTDS values for the links from the same subject versus those paired from different subjects. This procedure was performed for all pairs of systems and the identified links were assumed significant if the t-test *p*-value was $< 10^{-3}$. The significance threshold level was determined as the value above which all network links were statistically significant, which resulted in a threshold of 19% (Fig. 5).

TDS was also applied to the physiological data and the results were compared with CTDS.

#### Statistical analysis

In order to compare the number and strength of the links at different states, the Mann Whitney Wilcoxon (MWW) test was used (the distributions were not normal according to Jarque–Bera test).

## Conclusion

The new network physiology framework was proposed to generalise the TDS method, by quantifying pairwise directional coupling, while controlling for indirect links. The proposed framework outperformed the original and modified directional TDS as demonstrated in simulations. It was further applied in our cognitive function experiment, where different lateralised brain–heart-breath couplings were found during different cognitive states. It showed significantly higher number of interactions which were more consistent across subjects, during attention and alertness tests compared to rest. Our study addressed a significant gap in understanding the network physiology during cognitive states, which can be extended to future studies on cognitive dysfunctions and other applications.
